# I know what is missing here: electrophysiological prediction error signals elicited by omissions of predicted ”what” but not ”when”

**DOI:** 10.3389/fnhum.2013.00407

**Published:** 2013-07-29

**Authors:** Iria SanMiguel, Katja Saupe, Erich Schröger

**Affiliations:** BioCog, Institute for Psychology, University of LeipzigLeipzig, Germany

**Keywords:** predictive coding, Bayesian surprise, temporal orienting, predictive timing, self-generation, human, auditory cortex, efference copy

## Abstract

In the present study we investigated the neural code of sensory predictions. Grounded on a variety of empirical findings, we set out from the proposal that sensory predictions are coded via the top-down modulation of the sensory units whose response properties match the specific characteristics of the predicted stimulus (Albright, 2012; Arnal and Giraud, 2012). From this proposal, we derive the hypothesis that when the specific physical characteristics of the predicted stimulus cannot be advanced, the sensory system should not be able to formulate such predictions, as it would lack the means to represent them. In different conditions, participant's self-paced button presses predicted either only the precise time when a random sound would be presented (random sound condition) or both the timing and the identity of the sound (single sound condition). To isolate prediction-related activity, we inspected the event-related potential (ERP) elicited by rare omissions of the sounds following the button press (see SanMiguel et al., 2013). As expected, in the single sound condition, omissions elicited a complex response in the ERP, reflecting the presence of sound prediction and the violation of this prediction. In contrast, in the random sound condition, sound omissions were not followed by any significant responses in the ERP. These results confirmed our hypothesis, and provide support to current proposals advocating that sensory systems rely on the top-down modulation of stimulus-specific sensory representations as the neural code for prediction. In light of these findings, we discuss the significance of the omission ERP as an electrophysiological marker of predictive processing and we address the paradox that no indicators of violations of temporal prediction alone were found in the present paradigm.

## Introduction

The brain anticipates upcoming sensory stimulation. This has clear advantages, for example, we react faster and more accurately to predictable events (Anllo-Vento, 1995; Mangun, 1995), and we can detect them at lower thresholds (Hawkins et al., 1990; Luck et al., 1994; Correa et al., 2005). Prediction is intricately tied to attention, and the processing of predictable events in the brain is guided by the interaction between these two processes (Kok et al., 2012). Accordingly, sensory responses to predicted stimuli may be enhanced (e.g., when attending to an expected target, Mangun and Hillyard, 1991) or attenuated (e.g., when stimuli are self-generated Hesse et al., 2010; Timm et al., 2013), depending on their relevance for behavior. There is overwhelming empirical evidence that prediction has a pervasive influence on stimulus processing (for a review see Bendixen et al., 2012). However, it is still unclear exactly how predictions come about, and once a prediction has been formulated, what its neural representation code is. In other words, the neurophysiological basis for a variety of prediction effects is a matter of debate.

Several findings indicate that when a particular stimulus is strongly expected, brain activity in the particular areas coding for that stimulus type is modulated. For instance, functional brain imaging in attentional cuing tasks has shown that visual cortex activity is raised during expectancy of a visual target (Kastner et al., 1999). Electrophysiological studies have demonstrated that if a sound is omitted from a predictable pattern, an auditory-like response may be emitted (Raij et al., 1997; Hughes et al., 2001; Bendixen et al., 2009). A similar result is found in associative learning and conditioning studies: If two stimuli are repeatedly presented in close succession, the presentation of the first stimulus by itself can trigger responses that would usually require the presentation of the second stimulus (Den Ouden et al., 2009). Such anticipatory responses are also triggered for actions with predictable sensory consequences; for example when left and right button presses are paired with, respectively, the presentation of faces or houses, the button press alone can trigger activity in the corresponding content-specialized visual processing area (Kuhn et al., 2010). This collection of findings seems to indicate that strongly predicting a stimulus may trigger the activation of its neural sensory representation, much like what happens during imagination or memory retrieval (Albright, 2012).

This idea sits well with the theoretical and computational approach known as predictive coding (Friston, 2005), in which the predictive activation of sensory representations plays a fundamental role. In predictive coding, predictions are formulated in higher areas of the cortical hierarchy and are sent as top-down signals to lower areas, where they induce an expected pattern of activation. The lower area receives sensory input and contrasts it with the expected activity pattern. Any mismatch between the predicted pattern and that evoked by the input is sent to the higher area as prediction error. The same procedure is repeated in multiple hierarchical cortical levels, each area computing the difference between predictions received from the higher areas and the input received from the lower areas.

In sum, the evidence supports a model in which predictions are coded by selectively modulating the neural units whose response properties match the predicted stimulus' characteristics. However, humans are clearly also able to make unspecific predictions i.e., knowing that something will happen now but not knowing exactly what will happen. For example, we can certainly notice the difference between the adequate termination of a song and its undue interruption, even if we don't know exactly how the song would have continued otherwise. This kind of prediction is difficult to reconcile with the predictive coding models: If we do not have a specific sensory representation to predictively activate, then how do we predict?

In the present study we explore the neural code of sensory predictions by inducing a strong sensory prediction and unexpectedly omitting the predicted stimulus. Following predictive coding models, early sensory responses should equal the difference between the prediction and the input. For the particular case of omissions of predicted stimuli, since there is no input, the electrophysiological response observed should be an exact mirror image of the prediction, therefore giving access to its neural representational code (SanMiguel et al., 2013). We hypothesize that, if prediction indeed relies on the activation of stimulus-specific sensory representations, it should not be possible to generate predictions when the specific stimulus characteristics are unknown. Accordingly, in a situation in which we can only predict *when* a stimulus will be delivered but not precisely *what* stimulus it will be, no prediction error signals should be observed when the stimulus is omitted. Following our interrupted song example, if there is no identity prediction (we don't know how the song would continue) and no sensory input (the song stops), there should be no mismatch between the two and hence, no prediction error. If this hypothesis is correct, then it raises the additional question of how we can notice the undue interruption of the song.

## Materials and methods

### Participants

This experiment was conducted in accordance to the Declaration of Helsinki. All participants gave written informed consent for their participation after the nature of the study was explained to them. Fifteen healthy Leipzig University students (10 women, 5 men, 2 left-handed) ranging in age 19–34 years (mean = 24.1 years) volunteered to participate in the experiment. Participants either received course credits or were reimbursed for their participation. All participants had normal or corrected-to-normal vision, and reported no hearing impairment or history of psychiatric or neurological disease.

### Stimuli and procedure

The experimental task was delivered with Cogent 2000 v1.29 running on Matlab. Participants sat comfortably inside an electrically shielded chamber and fixated on a fixation cross displayed on a screen placed at a distance of approximately 100 cm from their eyes. In all conditions, participants pressed a button with the thumb of their dominant hand every 600–1200 ms on a cushioned Microsoft SideWinder Plug & Play Game Pad. In the sound conditions, button presses initiated the delivery of a sound on 87% of the trials (sound trials). Sounds were omitted on the remaining 13% of the button presses (omission trials). Omission trials were randomly placed with the restriction that the first five trials of each run of trials and the two trials immediately following an omission were always sound trials.

Sound stimuli consisted of 48 different common environmental sounds rated as identifiable by an independent sample of participants (see Wetzel et al., 2010). All sounds were shortened to have a duration of 200 ms, were tapered-cosine windowed (10 ms rise- and 10 ms fall-time), root mean square (RMS) matched and presented binaurally through headphones (Sennheiser HD 25-1). Participants wore soft foam earplugs during the whole experiment in order to silence any possible noise generated by the button presses. Prior to the start of the experiment, and with the earplugs inserted, participants adjusted sound volume to a loud but comfortable level while listening to the 48 sounds presented randomly with a stimulus onset asynchrony (SOA) of 800 ± 200 ms.

Two different sound conditions were performed. In the single sound condition, the same sound was presented in all trials of one block; hence both the timing and the identity of the sound could be predicted. A total of seven different sounds were used in this condition per participant, one sound per block. Across the whole participant sample, each of the 48 sounds was used in the single sound condition at least twice. In the random sound condition, a different sound was randomly selected in every trial out of the complete 48 sounds sample; hence only the timing but not the identity of the sound could be predicted. In addition to the sound conditions, a no-sound motor control condition was included in which no sounds were delivered after the button presses.

Prior to the start of the experiment, participants performed a short training without sounds to tune to the requested timing between button presses. During training, visual feedback on the timing between button presses was presented on every trial. Training could be repeated at any point during the experiment as needed if participants lost the pace. The different condition blocks were organized in pseudorandom order as follows. The experiment was divided in three parts. In the first part one no-sound motor control block, three single and three random sound condition blocks were performed in random order. In each the second and third parts, one no-sound motor control block and two blocks of each sound condition were performed in random order. Every block was ~3 min long. In total, 1386 sound trials and 203 omission trials were performed for each sound condition. A total of 600 trials were performed as no-sound motor control. Blocks could be repeated if an excessive number of trials fell outside the button-press timing limits enforced. Total experimental time was around 1 h 20 min.

### Electroencephalogram (EEG) acquisition

The EEG was continuously acquired at a sampling rate of 500 Hz from 64 Ag/AgCl active electrodes commonly referenced to the tip of the nose, the signal amplified by BrainVision Professional BrainAmp DC amplifiers and recorded with Vision Recorder v1.10 (Brain Products GmbH, Germany). Electrodes were mounted in an elastic cap (actiCAP, Brain Products GmbH, Germany) according to the 10% extension of the international 10–20 system (Chatrian et al., 1985). Three additional electrodes were placed in order to record eye movements, one electrode on the nasion and one below each eye (see Schlögl et al., 2007). The ground electrode was placed on the forehead.

### EEG preprocessing

EEG preprocessing was performed with EEGlab (Delorme and Makeig, 2004). Offline, the EEG was bandpass filtered from 1 to 100 Hz (windowed sinc FIR filter, Kaiser window, Kaiser beta 5.653, filter order 908), corrected for eye movements following Schlögl et al. (2007), and lowpass filtered (25 Hz lowpass, windowed sinc FIR filter, Kaiser window, Kaiser beta 5.653, filter order 908). Remaining artefacts were rejected by applying a 75μV maximal signal-change per epoch threshold. A −200 to +500 ms epoch was defined around each button-press. No baseline correction was applied, to avoid introducing motor preparation signals present in the baseline period into the post-stimulus waveforms (Urbach and Kutas, 2006). Epochs were averaged for each condition separately. All trials outside the 600–1200 ms button-press timing limits, the first five trials of each run of trials and the two sound trials immediately following an omission trial were excluded from analysis. On average 7.2% of the trials were rejected per condition (range 1–20.5%). These rejection rates resulted in a minimum of 145 omission trials per sound condition and 482 no-sound motor control trials included in the final averages per participant.

### Event-related potential (ERP) analysis

**Figure 1:**
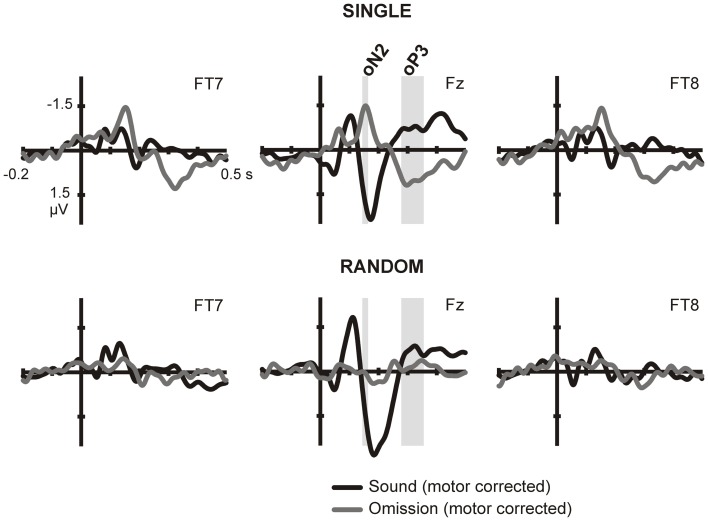
**ERPs in sound and omission trials**. Sound and omission responses in the single sound **(top)** and random sound **(bottom)** conditions, plotted for one selected electrode in each ROI (temporal left: FT7, frontocental midline: Fz, temporal right: FT8). Both sound and omission responses are motor-corrected via subtraction of the no-sound motor control waveform. Clear omission-related responses are present only for omissions in the single sound condition and not for omissions in the random sound condition. The analysis time-windows for the oN2 (144–164 ms) and oP3 (278–356 ms) components are indicated with gray shading on the midline ROI electrode.

The presence of prediction-related activity in each sound condition was first verified comparing omissions trials to the physically equivalent silent button presses in the no-sound motor control condition, where no prediction should be present. To identify time-windows and regions of interest for this comparison, we combined a priori knowledge with an assumption free, cluster-based random permutation procedure. The analysis was constrained by a priori knowledge on the sequence of responses elicited by single sound omissions in a recent study. Following SanMiguel et al. (2013), we expected a series of three consecutive omission responses (omission N1, N2, and P3): a first negative response, present over frontotemporal scalp locations in the time period between 0 and 100 ms, followed by a second negative response between 100 and 200 ms, maximal over the frontocentral midline and finally a broadly distributed positive deflection between 200 and 400 ms. Hence, the statistical analysis focused on three regions of interest (ROIs): left temporal (FT7, FC5, T7, C5), frontocentral midline (Fz, FCz, Cz) and right temporal (FC6, FT8, C6, T8). Given this a priori information, the omission N2 and P3 could be clearly identified on the grand-average single sound omission waveforms. Thus, time windows of interest for these two components were defined around the deflection peaks on the frontocental midline electrodes (oN2, 144–164 ms; oP3, 278–356; see Figure 1). Statistical analyses for these two components were carried out on the mean amplitude over the defined time-windows and over all electrodes in each ROI. For the oN2, amplitude measures on the frontocentral midline ROI were contrasted with a two-sided, paired samples *t*-test between the omission trials and the no-sound motor control, separately for the single and random sound conditions. For the oP3, the presence of responses was tested with a condition (omission trials, no-sound motor control) × region (left temporal ROI, frontocentral midline ROI, right temporal ROI) ANOVA, separately for the single and random sound conditions.

The time window for the oN1, however, could not be clearly identified by the same procedure, as a slow rising negativity was present on the temporal ROIs over the whole 0–100 ms time-period (see results). For this reason, a cluster-based non-parametric permutation testing procedure implemented in the Fieldtrip toolbox (Oostenveld et al., 2011) was applied on the time-courses of the electrodes included in the temporal ROIs, in order to identify clusters of interest in the time domain. This procedure follows the approach described in Maris and Oostenveld (2007). Essentially, the time-course of the two conditions was compared with a point by point dependent samples *t*-test and clusters of adjacent significant points (*p* < 0.1) were identified. For each cluster, a cluster-level statistic was calculated by taking the sum of all the individual *t*-statistics within that cluster. The multiple comparisons problem was solved using non-parametric testing at the cluster level. A comparison distribution was generated by randomly permuting the values between conditions 1000 times, and computing the cluster-level statistic in each of the permutations. A cluster was considered significant if the probability of observing a larger cluster level statistic from the shuffled data was below 5%. The single and random omission ERPs were compared to the motor control ERPs following this procedure to identify significant temporal clusters, particularly in the early (0–100 ms) time-window. On the basis of the cluster analysis, the time-window for the omission N1 was defined (oN1, 42–92 ms), and an additional earlier time-window of interest was identified, i.e., the early negativity window (eNeg, −20 to 40 ms; see results for a closer description of the selection procedure for these two windows). Confirmatory parametric testing was additionally carried out on the mean amplitude in these windows in the temporal ROIs. Separate condition (omission trials, no-sound motor control) × hemisphere (left temporal ROI, right temporal ROI) analyses of variance (ANOVAs) were performed for the random and single sound conditions in the oN1 and eNeg time-windows.

After the presence of prediction-related activity for omission trials was tested in each sound condition, omission trials were directly contrasted between the single and random sound conditions in each of the time-windows of interest. In the oN1 and eNeg time windows, amplitude measures for the single and random sound omissions were contrasted with a condition (single omission, random omission) × hemisphere (left temporal ROI, right temporal ROI) ANOVA. In the oN2 time window amplitude measures on the frontocentral midline ROI were contrasted with a two-sided, paired samples *t*-test between the omission responses of the single and random sound conditions. Finally, in the oP3 time window, differences between omission responses in the single and random sound conditions were tested with a condition (single omission, random omission) × region (left temporal ROI, frontocentral midline ROI, right temporal ROI) ANOVA.

### Topographic analysis

ERP voltage distributions for the eNeg, oN1 and oN2 ERP components were transformed into scalp current density maps (SCD) following the method described in Perrin et al. (1989). SCD maps are reference free and indicate scalp areas where current lines emerge from or converge into the scalp, allowing an easier visual estimation of the underlying generators than scalp potential maps. For SCD analyses, the maximum degree of the Legendre polynomials was chosen to be 50, and the order of splines (m) was set to 4. A smoothing parameter of 10^−4^ was applied.

### Source analysis

Brain sources for the relevant ERP responses were estimated performing brain electrical tomography analyses, using the Variable Resolution Electromagnetic Tomography (VARETA, Bosch-Bayard et al., 2001) approach. With this technique, sources are reconstructed by finding a discrete spline-interpolated solution to the EEG inverse problem: estimating the spatially smoothest intracranial primary current density (PCD) distribution compatible with the observed scalp voltages. This allows for point-to-point variation in the amount of spatial smoothness and restricts the allowable solutions to the gray matter, based on the probabilistic brain tissue maps available from the Montreal Neurological Institute (Evans et al., 1993). This procedure minimizes the possibility of “ghost sources,” which are often present in linear inverse solutions (Trujillo-Barreto et al., 2004). A 3D grid of 3244 points (voxels, 7 mm grid spacing), representing possible sources of the scalp potential, and the recording array of 64 electrodes were registered with the average probabilistic brain atlas developed at the Montreal Neurological Institute. Subsequently, the scalp potential in the latency range of the relevant components was transformed into source space (at the predefined 3D grid locations) using VARETA. Statistical parametric maps (SPMs) of the PCD estimates were constructed based on a voxel by voxel Hoteling *T*^2^ test between conditions in order to localize the sources of the response. For all SPMs, Random Field Theory (Worsley et al., 1996) was used to correct activation threshold for spatial dependencies between voxels. Results are shown as 3D activation images constructed on the basis of the average brain.

## Results

Participants were able to maintain a stable pace between button presses keeping an average of 784 ± 46 (SD) ms between presses in the random sound condition, 805 ± 44 ms in the single sound condition and 818 ± 50 ms in the no-sound motor control. A mean of 1.3 (range: 0–3) blocks were repeated per participant.

To isolate prediction-related activity we compared electrical brain responses time-locked to button presses resulting in physically identical stimulation (i.e., no sound was delivered), but differing in the degree of prediction for upcoming sounds. No prediction for a sound should be present in blocks in which button presses never caused a sound (no-sound motor control condition). In contrast, highly precise predictions about the forthcoming sound could be formulated during the single sound condition, while only predictions about the sound onset should be generated during the random sound condition. Thus, any activity elicited to omissions of single and random sounds above the no-sound motor control responses was considered a neural reflection of prediction. Sound and omission responses for each of the sound conditions are depicted on Figure 1. In order to identify the sound- and omission-related responses, motor activity has been subtracted from the waveforms. Thus, the plots show the difference between the sound- or omission-related potential in the sound conditions and the response in the no-sound motor control. The ERPs show clearly identifiable omission responses in the single sound condition, when a specific prediction could be formulated, while a less consistent pattern of activity is visible in the random sound condition.

In the single sound condition, large deflections corresponding to the oN2 and oP3 components can be observed, and corresponding analysis time-windows were defined around these two peaks on the central midline ROI. Statistical analysis carried out for these two components, corroborated the presence of significant omission responses in the single sound condition [oN2: *t*_(14)_ = −4.086, *p* = 0.001; oP3: *F*_(1, 14)_ = 29.124, *p* < 0.001] but not in the random sound condition [oN2: *t*_(14)_ = 0.255, *p* = 0.802; oP3: *F*_(1, 14)_ = 0.190, *p* = 0.670]. In the random sound condition, a significant condition × ROI interaction was found for the oP3 time-window [*F*_(2, 28)_ = 10.043, *p* = 0.002]; however, *post-hoc t*-tests in each ROI corroborated that there was no significant response elicited in any of the ROIs [temporal left: *t*_(14)_ = 1.104, *p* = 0.288; temporal right: *t*_(14)_ = 1.970, *p* = 0.095; midline: *t*_(14)_ = −1.253, *p* = 0.231]. The direct statistical contrast between responses elicited in omission trials in the single and random sound conditions corroborated the presence of larger prediction-related activity for omission trials in the single sound than in the random sound condition in both time windows [oN2: *t*_(14)_ = −4.813, *p* < 0.001; oP3: *F*_(1, 14)_ = 17.453, *p* = 0.001]. Again a significant interaction between condition and ROI was found for the oP3 time-window [*F*_(1, 14)_ = 8.805, *p* = 0.006]. Nevertheless, *post-hoc* paired comparisons corroborated that omission trials in the single and random sound conditions differed significantly in all ROIs [temporal left: *t*_(14)_ = 4.849, *p* < 0.001; temporal right: *t*_(14)_ = 3.331, *p* = 0.005; midline: *t*_(14)_ = 4.070, *p* = 0.001].

**Figure 2:**
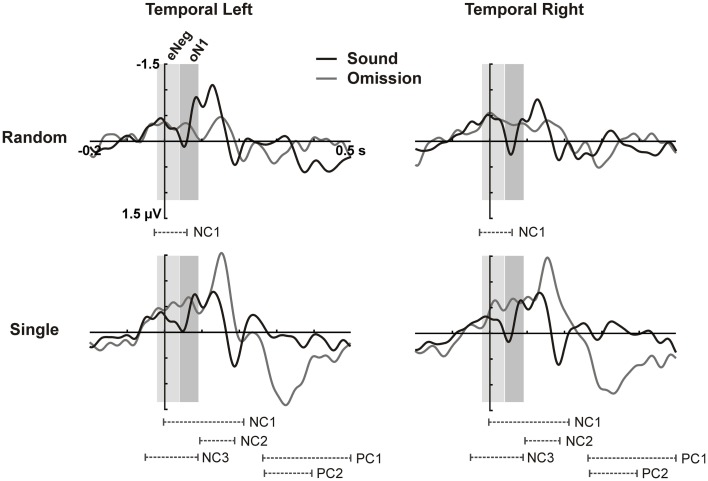
**Cluster analysis on temporal ROIs**. ERPs for the temporal left **(left column)** and temporal right **(right column)** ROIs (averaged over electrodes in the ROI) are plotted for sounds and omissions in the random sound **(top row)** and the single sound **(bottom row)** condition. Both sound and omission responses are motor-corrected via subtraction of the no-sound motor control waveform. Temporal clusters in which omission responses differed significantly from the motor control are indicated under the waveforms for each sound condition (NC: Negative cluster, PC: Positive cluster). Time-windows defined on the basis of the cluster analysis for the eNeg (−20 to 40 ms) and oN1 (42 to 92 ms) components are indicated with gray shading.

In the time period between 0 and 100 ms, we expected to identify the oN1 component on the temporal ROIs; however, no clear peak can be observed in this time period but rather a sustained negativity, starting even before 0 ms (see Figure 1). Therefore, we adopted a data-driven approach to identify additional time periods of interest where the sound omission waveforms significantly differed from the motor control, particularly in the early time period. The results of the cluster-based permutation test are depicted in Figure 2. In the single sound condition, significant clusters were found corresponding to the oP3 (positive cluster 1 [PC1], 266–500 ms, *p* < 0.001 and PC2, 270–398 ms, *p* < 0.001) and the oN2 (negative cluster 2 [NC2], 94–184 ms, *p* = 0.008) components. Additionally, two significant clusters were found in the early time period. NC1 [−2 to 214 ms, *p* < 0.001] covers most of the sustained negativity and the oN2 response, and NC3 (−52 to 92 ms, *p* = 0.008) covers the earlier part of the sustained negativity. In the random sound condition, a single significant cluster was found (NC1, −28 to 60 ms, *p* = 0.041). This cluster covers approximately the same time-period as NC3 in the single sound condition, and similar deflections are observable in the omission and sound waveforms of both conditions in this time window. Thus, to be able to characterize the topographies and sources of this early negativity, a representative time-window was defined (eNeg, −20 to 40 ms), centered around the coinciding portions of the significant clusters (NC1, random condition and NC3, single condition) and covering the peak of the deflection in both conditions. Finally, the oN1 time-window was identified in the time period covered by NC1 in the single sound condition, which was not included in the eNeg or the oN2. In this time-window, a differentiated deflection can be identified in the omission waveforms of both the single and the random sound conditions, thus a representative time-window was defined around this peak (oN1, 42–92 ms). Consistent with SanMiguel et al. (2013), the oN1 time-window also includes the Na component of the T-Complex elicited by sounds.

Statistical analyses carried out on the eNeg and oN1 time-windows in the temporal ROIs corroborated the results of the cluster-based analysis. In the eNeg time-window, the omission waveforms differed significantly from the motor control in both the random [*F*_(1, 14)_ = 9.609, *p* = 0.008] and the single [*F*_(1, 14)_ = 9.808, *p* = 0.007] sound conditions, while the random and single sound omission waveforms did not significantly differ from each other [*F*_(1, 14)_ = 0.387, *p* = 0.544]. In the oN1 time-window, a significant response was only elicited by single sound omissions [*F*_(1, 14)_ = 11.208, *p* = 0.005] and not by random sound omissions [*F*_(1, 14)_ = 3.741, *p* = 0.074], although a trend was apparent. The direct comparison between single and random sound omissions in the oN1 time-window corroborated the presence of a larger response in the single than in the random condition [*F*_(1, 14)_ = 6.592, *p* = 0.022].

**Figure 3:**
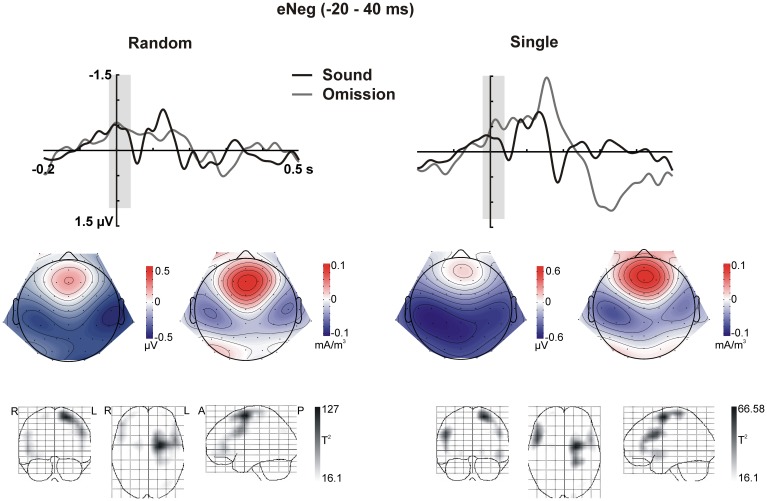
**Early Negativity (eNeg, −20 to 40 ms)**. The response elicited by sound omissions in the eNeg window is depicted for the random sound (left) and single sound (right) conditions. **Top row:** ERPs for the temporal right ROI (averaged over electrodes in the ROI) are plotted for sounds and omissions. Both sound and omission responses are motor-corrected via subtraction of the no-sound motor control waveform. The time window defined for the eNeg is indicated in gray shading. **Middle row:** Scalp potential (left) and scalp current density (right) maps for the motor-corrected omission response in the marked eNeg window. **Bottom row:** Statistical parametric maps of the VARETA source estimation for the sound omission vs. motor control contrast in the eNeg time-window. Maps are thresholded at *p* < 0.001. The solution maximum for both the single and the random conditions is located at Talairach coordinate *x* = −21, *y* = −4, *z* = 63, corresponding to superior frontal gyrus (SFG), Brodmann area 6 (BA6).

**Figure 4:**
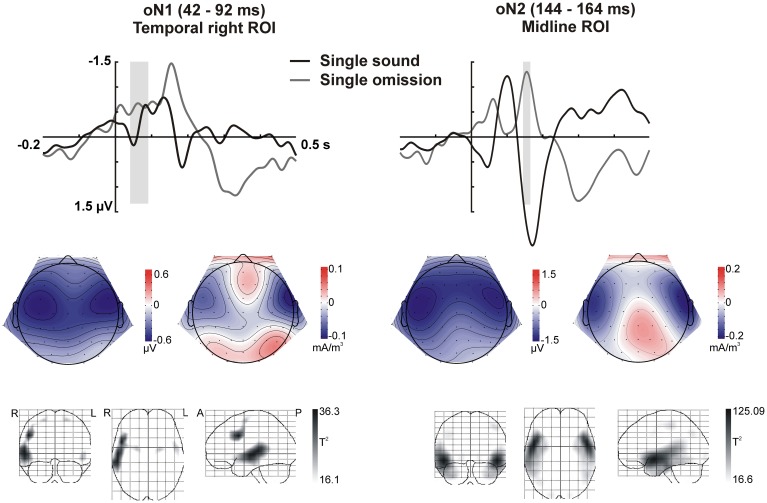
**Omission N1 (oN1, 42–92 ms) and omission N2 (oN2, 144–164 ms) responses in the single sound condition**. The response elicited by sound omissions in the single sound condition in the oN1 (left) and oN2 (right) time-windows is depicted. **Top row:** ERPs for the temporal right (left) and midline (right) ROIs (averaged over electrodes in the ROI) are plotted for sounds and omissions. Both sound and omission responses are motor-corrected via subtraction of the no-sound motor control waveform. The time-windows defined for the oN1 (left) and oN2 (right) are indicated with gray shading. **Middle row:** Scalp potential (left maps) and scalp current density (right maps) maps for the motor-corrected omission response in the marked oN1 and oN2 time-windows. **Bottom row:** Statistical parametric maps of the VARETA source estimation for the single sound omission vs. motor control contrast in the marked oN1 and oN2 time-windows. Maps are thresholded at *p* < 0.001. The solution maximum for the oN1 time-window is located at Talairach coordinate *x* = 50, *y* = 10, *z* = 34, corresponding to right middle frontal gyrus (rMFG), Brodmann area 9 (BA9). A second, larger cluster of activity extends throughout large portions of the superior and middle temporal gyrus (rSTG, rMTG), including Brodmann areas 21 and 22. The solution maximum for the oN2 time-window is located at Talairach coordinate *x* = 50, *y* = 3, *z* = −10, corresponding to right superior temporal gyrus (rSTG). Brodmann areas 38, 22, and 21 in STG are also located within a 4 mm range of the solution maximum.

In sum, the statistical analysis of the ERP waveforms allowed the identification of a series of responses in the omission waveforms. When participants expected to hear a sound after pressing the button, an early negativity was present at the moment of the button press (eNeg), irrespective of whether a specific or a random sound was expected. Subsequent prediction-related activity however, was only present in the single sound condition, when a specific sound was expected. In this condition, when the predicted sound was omitted, the early negativity was followed by the omission N1, N2, and P3 responses. The scalp distribution and VARETA source estimation for each of these components are characterized in Figure 3 (eNeg, single and random condition) and Figure 4 (oN1, oN2, single condition). The early negativity (eNeg, Figure 3) shows consistent topographies and sources in both the random and single sound conditions, indicating a probable motor origin. The maximum of the source estimation is located in premotor/supplementary motor areas in the left hemisphere. Given that thirteen out of fifteen participants were right-handed, the lateralization is on average contralateral to execution hand. In the single sound condition, both the oN1 and oN2 responses (Figure 4) show a scalp distribution consistent with sources in auditory cortices. The VARETA source estimation yielded similar sources on superior temporal gyrus (STG) for both the oN1 and oN2, with the oN1 showing a more posterior and right-lateralized distribution compared to the oN2. In the oN1 time-window, the VARETA solution also shows a significant source of activity in the right middle frontal gyrus (rMFG).

## Discussion

In the present study, we tested a hypothesis on the neural substrate for sensory prediction. We hypothesized that when participants expect to hear a specific sound after pressing a button, the button press triggers the predictive activation of the sound's representation in auditory cortex. Unless prediction is present, if no auditory stimulation is presented, no auditory sensory responses should be observed. Hence, we inspected brain responses elicited when the self-generated sounds were omitted after the button press. Any electrophysiological responses elicited by sound omissions should be a direct consequence of predictive activity. Following predictive coding models, we assume that early sensory responses reflect the informational difference between sensory prediction and sensory input. Given that in omission trials there was no input, in this particular case the difference between prediction and input should directly reflect the neural code of the sensory prediction. Therefore, examining early responses obtained in omission trials should help answer the question of how prediction is represented. If predicting the occurrence of a particular sound is accomplished by modulating the sensory units that form the sound's sensory representation, sound omissions should trigger a mismatch between prediction and input only in those modulated units, causing them to respond, and eliciting an auditory response. However, if the specific physical characteristics of the predicted sound are unknown, the sensory system should not be able to predict it, as it would have no means to represent it. Accordingly, in this case, if the sound is omitted, no prediction-related responses should be observed. The results support this line of reasoning: when participant's button presses always generated the same sound, sound omissions elicited an auditory-like response, followed by subsequent error signals. Conversely, when button presses generated a different sound on every trial, the sound omission did not elicit any significant auditory responses in the ERP and subsequent error signals were also not observed. However, in both cases the motor plan appeared to carry unspecific expectation activity.

Electrophysiological activity in premotor areas contralateral to the execution hand differed at the moment of the button press depending on whether this motor act was expected to have an associated auditory consequence or not. In both the random and the single sound condition, we observed an enhanced negativity around the time of the button press, compared to the no sound motor control. Pleasingly, this negativity was present irrespective of whether the sound was later omitted or not (cf. Figures 2, 3), as at this moment the sound had not yet been presented either way. The effect was also identical irrespective of whether a specific sound could be predicted or not, and within this time-window, no prediction-related activity was observed in auditory areas. These facts indicate that the early negativity does not represent a specific sensory prediction, but rather some form of expectation associated to the motor act, which does not carry specific information about the predicted stimulation. These characteristics fit well with the possibility that we are observing an efference copy of the motor command. According to motor control models, whenever a motor act is planned, a copy of the motor plan (i.e., the “efference copy”) is generated and sent to sensory processing areas, where the sensory consequences of this motor command can be anticipated (Crapse and Sommer, 2008). In this way, intended and achieved effects of our motor commands can be compared, providing necessary feedback for achieving efficient motor control (Wolpert and Ghahramani, 2000). Alternatively, differences in the magnitude of motor activity could be due to different amounts of attention being invested in the motor act in motor only and motor auditory blocks.

Prediction-related responses showing specificity for the predicted stimulation were only observed in the single sound condition. When a specific sound was expected but it was not delivered, the earliest response observed in the ERPs at the time of the sound omission was a fronto-temporal N1 portraying typical characteristics of an auditory N1 response, including sources which localized to auditory cortex. Thus, we propose that the N1 response elicited by sound omissions represents the prediction of the particular sound (see also SanMiguel et al., 2013). Moreover, the elicitation of the N1 response signals that this prediction was not matched by the input, and thus it was erroneous. The computation of the prediction error is accomplished within sensory cortex, which presumably feeds forward this information to higher cortical areas. The N1 response was followed by an anterior N2 and a P3 response. Anterior N2s are typically elicited in paradigms which tap into at least one of two core concepts: the presence of deviance or the presence of errors or conflict in the context of action monitoring (Folstein and Van Petten, 2008). These two characteristics both play a defining role in omission trials in the present paradigm: omissions were rare events, which consisted in motor acts that did not have the expected consequences, therefore indicating a possible action error. Indeed, previous studies in which actions were paired with unexpected or unintended outcomes have reported similar anterior N2 responses (Gehring et al., 1993; Falkenstein et al., 2000; Katahira et al., 2008; Gentsch et al., 2009; Iwanaga and Nittono, 2010). As in the present study, the N2 is often followed by a P3, forming the N2-P3 complex. The P3 response is thought to reflect attention orienting triggered by surprising events (Friedman et al., 2001; Escera and Corral, 2007) and the updating of mental models to integrate new information (Barceló et al., 2006; Polich, 2007). In line with these ideas, we propose that when a sound omission was encountered, first, a prediction error was detected, signaled here by the N1 response. This error in prediction characterizes the omission as an unexpected and thus surprising event, leading to a mobilization of attentional resources to process the event in depth. Further processing was signaled by the N2-P3 complex, which reflects cognitive control measures related to the evaluation of the error's significance in context, and the adjustment of the forward model that generated the prediction in order to minimize future error.

None of these prediction-related responses were observed when a random sound was omitted. The failure to elicit an N1 response fits to our hypothesis regarding the neural representation of prediction. In this condition, no specific sound representation could be modulated in sensory cortex, hence there was no difference between the (lack of) prediction and the (lack of) input and no N1 was elicited. More surprisingly, the N2-P3 complex was also absent. One could suspect that even when a specific sound cannot be predicted, participants can still notice the absence of sound as a rare event and that this would trigger similar evaluation and cognitive control processes as when a specific sound is expected. That is, that even when a prediction error response can't be elicited in early sensory cortex, parallel routes might exist to trigger higher cognitive processing of the omission. In fact, differences in motor activity at the time of the button press do indicate that some form of expectation was present also in this condition. However, there seemed to be no consequences when this expectation was violated, as no error responses were elicited in this case. Thus, the present findings argue for a serially organized system and imply that, as long as specific identity predictions cannot be formulated, deviating events are not evaluated in depth and do not trigger attentional orienting and cognitive control measures. Although somewhat puzzling, this finding is consistent with psychological studies on what people consider a surprising event. Teigen and Keren (2003) showed that the same unusual event can be rated as more or less surprising in different scenarios, depending on whether there is one highly probable alternative or the alternative possible events are also each relatively improbable. Maguire et al. (2011) have proposed that, rather than being a direct function of the probability of the event, subjective ratings of surprise depend on the ease with which the event can be integrated into an existing explanatory model. This proposal is conceptually similar to Itti and Baldi's formal Bayesian definition of surprise (Itti and Baldi, 2009; Baldi and Itti, 2010). Bayesian surprise quantifies how incoming data affects an observer, by measuring the difference between the observer's beliefs before and after receiving the new data. New data that is difficult to integrate into the current explanatory model (i.e., the observer's beliefs) requires that significant changes are made to the model, thus yielding a high value of Bayesian surprise. This perspective stresses the importance of the observer's beliefs: when the observer cannot make confident predictions, any event holds little surprise value, no matter how improbable it is by itself.

Hence, the absence of the N2-P3 complex for omissions in the random sound condition might be related to a low level of surprise in this scenario. The P3 response has historically been tied to the concept of surprise (Sutton et al., 1965; Donchin, 1981), and more recent studies have been able to model trial-by-trial fluctuations in P3 amplitude using various estimations of surprise values (Mars et al., 2008; Kolossa et al., 2012). However, alternative models have rather stressed the model updating aspects of P3-eliciting events. Bayesian surprise neatly encompasses both aspects. Thus, our findings are consistent with this framework and provide a possible neurophysiological basis for the computation of surprise values. In particular, the surprise value of an improbable event might critically depend on the sensory system's capabilities for representing the observer's beliefs. If the expected event is beyond the representational capabilities of low-level sensory cortices (e.g., when it is a category of stimuli that do not share any one particular physical property), then the new data (in this case the random sound omission) would encounter no model to modify, and so it would generate a low value of Bayesian surprise.

Nevertheless, it is quite unlikely that prediction did not play any role in the processing of self-generated sounds in the random sound condition. First of all, as discussed above, motor activity was altered when participants expected the button press to have an auditory consequence. Moreover, outside the laboratory, predictions can hardly ever be made with absolute certainty about the precise physical characteristics of the stimulus. Further, there is some evidence that stimulus processing is modulated by temporal predictability regardless of whether the specific identity of upcoming stimuli can be predicted (Baess et al., 2008; Lange, 2009). Possibly, a relatively unspecific prediction could still be formulated in auditory cortices in the random sound condition, based on the few available predictable physical characteristics of the upcoming stimulus (e.g., its sensory modality, spatial location). Moreover, neural units with rather unspecific response properties are present in sensory cortices, including auditory (Jones, 1998). These unspecific units could arguably be recruited to represent imprecise predictions. While there was no significant prediction-related activity in the omission ERPs of the random sound condition in the oN1, oN2, and oP3 windows, it is worth noting that the random omission time courses do not appear to be random noise either. There are visible, albeit quite small deflections for random omissions in each of these time-windows (see Figure 2) which are consistent with the responses found in the single sound condition also in their scalp topography (data not shown). According to predictive coding models, the responsiveness of prediction error units (i.e., sensory units) is weighted by the precision of the available prediction (Feldman and Friston, 2010). Hence, the lack of significant prediction-related activity in the random sound condition could be partly explained by the low precision of the prediction in this condition, leading to a down-weighting of the prediction error response. Additionally, in the random sound condition, omissions almost exclusively incur a violation of temporal prediction. The mechanisms of temporal prediction are different from those of identity prediction. According to current models, temporal prediction is a strictly modulatory process that relies on the generation of ideal windows for stimulus processing, coinciding with the occurrence of the relevant stimuli (Large and Jones, 1999; Schroeder and Lakatos, 2009). In these temporal windows, the neural responsiveness of sensory areas is increased so that stimuli arriving at the predicted point in time receive privileged processing. Therefore, temporal prediction by itself is not expected to drive any responses if no stimulation is presented, as is the case of omission trials. The present paradigm is especially sensitive to this distinction.

Temporal prediction has been mostly investigated by comparing responses to task-relevant stimuli presented at expected vs. unexpected moments in time (see Nobre et al., 2007). Typically, in these studies, the physical characteristics of the stimuli are always highly predictable. Nevertheless, a few studies have been able to show that temporal prediction by itself has no effects on early perceptual processing of task-relevant visual stimuli (Miniussi et al., 1999; Griffin et al., 2002), but has a multiplicative effect when combined with identity prediction (Doherty et al., 2005). In a revision of these findings, Nobre et al. (2007) conclude that perceptual influences of temporal expectations may be dependent upon other receptive-field properties of neurons. In other words, that temporal prediction can only have a modulatory effect on identity predictions that low-level sensory cortices are able to represent. Omission responses are particularly suited to investigate this claim, given that there is no input to be modulated, hence all activity is purely a reflection of predictive processes. However, temporal orienting studies investigating stimulus omissions are scarce. In a rare example, Langner et al. (2011) found greater omission-related responses in basal ganglia for specific compared to non-specific expectations when temporal prediction was held constant. The authors related basal ganglia activity to Bayesian surprise, but effects on sensory cortices were not reported.

In conclusion, the present findings support a model in which identity predictions are accomplished via forward modeling, producing a template of the predicted stimulus and making use of the receptive field properties of low-level sensory units to represent it; while temporal prediction has a strictly modulatory effect, boosting the responsiveness of the sensory units that hold the identity predictions, within the time windows in which stimuli are expected. As a consequence, the present findings indicate that electrophysiological responses commonly associated with prediction error signaling (N2, P3), and the attentional and cognitive control processes associated with them, are only elicited by violations of identity prediction. These findings raise important questions regarding the role of temporal expectation. In particular, it is unclear how violations in purely temporal (without identity) expectations can be detected.

### Conflict of interest statement

The authors declare that the research was conducted in the absence of any commercial or financial relationships that could be construed as a potential conflict of interest.
